# Development and initial validation of the addressing client needs with social determinants of health scale (ACN: SDH)

**DOI:** 10.1186/s12913-023-09292-z

**Published:** 2023-04-19

**Authors:** Kaprea F. Johnson

**Affiliations:** The Ohio State University, 281 W Lane Ave, Columbus, OH USA

**Keywords:** Social determinants of health, Competency scale, Health service professionals, Healthcare management, Exploratory factor analysis, Confirmatory factor analysis

## Abstract

**Background:**

The purpose of this study was to validate a scale that can be used by healthcare service professionals, healthcare systems, educators, and researchers to assess health service professionals’ social determinants of health (SDOH) competency; with competency defined as their knowledge, awareness-biases, skills, and actual preparedness to address SDOH challenges.

**Methods:**

An Exploratory Factor Analysis (EFA) was conducted with a sample of 220 health service professionals, and 6 factors were identified. A Confirmatory Factor Analysis (CFA) was conducted with 303 health service professionals and the 6-factor solution was supported, with 22 items.

**Results:**

The reliability estimates for the 6 factors are as follows: Factor 1, *Action Toward Addressing SDOH* (*a* = .85); Factor 2, *SDOH Knowledge (a* = *.94);* Factor 3, *Negative Attitude toward Addressing SDOH* (*a* = .79); Factor 4, *Systemic Accountability* (*a* = .81); Factor 5, *School Preparation* (*a* = .86); and Factor 6, *Perception of the Cause of SDOH* (*a* = .94).

**Conclusion:**

The ACN:SDH scale is the first validated measure that can be used to systematically appraise health service professionals’ SDOH competency.

**Supplementary Information:**

The online version contains supplementary material available at 10.1186/s12913-023-09292-z.

## Background

The social determinants of health (SDOH) are the conditions in which people are born, grow, live, work, and age, and the wider set of forces and systems shaping the conditions of daily life [1]. The SDOH impacts patient health by contributing to health disparities and overall poor health which raises healthcare costs and impacts the healthcare organization [2, 3]. In addition, delivering high-quality healthcare entails health service professionals’ understanding of the SDOH needs of patients and the communities in which health services are delivered [3]. Health service professionals must have SDOH competence to identify and address patients at risk of poor health outcomes due to SDOH [4, 5]. Competence includes being knowledgeable, feeling prepared, having the skills, and understanding the resources available to address SDOH needs in practice [6]. The purpose of the present study was to develop and validate a scale for appraising health service professionals’ competence in addressing SDOH challenges in practice. Of note, in the current study, health service professionals is a term used to represent both behavioral health (i.e., specialists who provide mental health and/or substance use care or treatment) and medical health (i.e., specialists who provide medical care or treatment). A scale of this nature can be used by healthcare systems to strategically develop a workforce able, prepared, and competent to address SDOH.

### Competence

SDOH competence and understanding the relationship to health and wellbeing is critical for a practitioner to address SDOH needs in practice [6, 7]. Research has shown that physicians continue to lack sufficient training in SDOH, which minimizes their ability to address SDOH needs in practice [6]. In a qualitative study of pediatric nurse practitioners, observers found that the trainees were able to identify some social health needs but very few intervened [8]. Feedback from the two cohorts of pediatric nurse practitioners (*N* = 36) found that trainees expressed discomfort in asking SDOH questions that they deemed sensitive and they experienced difficulty in negotiating their priority to address the medical need versus the non-medical social health need [8]. In a study of civil servants (*N* = 153), researchers found that only 50% of the respondents were familiar with SDOH, 83% indicated they need more information on effective intervention strategies [9]. Similarly, another study in Canada investigated the relationship between understanding and attitudes toward SDOH with people who worked at community-based organizations [10]. Results found that support for addressing health inequalities was associated with increased awareness of SDOH and a broad view of the influence of SDOH on overall health and wellness [10].

Lastly, healthcare systems are eager to effectively address SDOH needs of patients because of the positive impact on population health, healthcare costs, and overall patient experiences [11]. To improve health equity and minimize health disparities, healthcare systems are applying frameworks for addressing SDOH, engaging in community collaboration and cross-sector partnerships, and using technology for interventions [11–14]. However, workforce readiness to address SDOH is a primary driver of program success and better patient outcomes [15]. To address workforce readiness, there is a need for a scale to measure provider SDOH competence. Currently, no known scales assess health service professionals’ competence to address SDOH in practice. To date there is an overreliance on qualitative assessments of SDOH knowledge, however, these studies are not generalizable to a larger population of health service professionals [16, 17]. The closest available Likert scale was developed to assess knowledge of the SDOH framework and understanding in five areas of empirical interests: awareness, understanding, and attitudes toward the SDOH framework [18]. The scale was a part of a larger project to evaluate health and social service systems however, the scale could not be validated or located in its entirety [18]. There were no other available Likert scales to assess SDOH competence for health service professionals known to the author at the time of this study’s development in 2019.

### Measurement instrument theoretical foundations

Competence is related to education and learning, therefore, to understand health service professionals’ SDOH competence the scale developed is based on a model of interprofessional education created by the Institute of Medicine (IOM) committee on measuring the impact of interprofessional education on collaborative practice and patient outcomes [19]. In addition, because addressing SDOH is aligned with cultural competence and social justice; the awareness, knowledge, and skills structure of the Multicultural Counseling Competencies and Standards was also utilized [20, 21]. The IOM model has four interrelated components that relate to overall health and system outcomes. The four interrelated components include (1) a learning continuum; (2) the outcomes of learning; (3) individual and population health outcomes; (4) system outcomes such as organizational changes, system efficiencies, and cost-effectiveness; and the major enabling and interfering factors that influence implementation and overall outcomes [19]. The ACN: SDH focuses on the outcomes of learning, in the model which includes attitudes/perceptions, knowledge/skills, collaborative behavior, and performance in practice [22]. The learning continuum (i.e., foundation and graduate education, and continuing professional development) should inform learning outcomes and impact the health care system along with patients; of specific interest to the current researcher is impacting individual health and population/public health. A key component to impacting individual health is understanding the gaps in learning outcomes for health service professionals’ (i.e., SDOH competence; IOM, 2015, p. 28) [15].

### The current studies

The purpose of the present studies was to develop and validate an SDOH competency assessment for health service professionals. Competence is related to education and learning, and the scale is developed based on a model of interprofessional education created by the Institute of Medicine (IOM) [15] along with the Multicultural Counseling Competencies and Standards [23, 24]. The research questions include: (1) What is the factor structure and reliability of the ACN: SDH? (2) Is the emergent factor structure of the ACN: SDH confirmed in a second sample?

## Methods

### Procedures

Data collection began after IRB approval in Fall of 2019 for Study 1(EFA) and Spring of 2020 for Study 2 (CFA); these were two separate recruiting events. Participants were recruited using an email list of health service professionals and through health professional organization listservs. The following inclusion criteria were used to recruit and select participants for the study: (1) Foundation education in a healthcare field, (2) Professional identity as a healthcare provider, (3) Experience with healthcare practice as a provider, and (4) over 18. The informed consent from study participants was written. The survey started with a consent form that described the purpose of the study and associated risk, followed by the demographic questionnaire and the scales.

### ACN: SDH instrument development and rigor

Best practice guidelines for scale development were followed, including developing an initial set of items and checking face validity using an expert panel [25]. An initial set of 163 items was created based on the theoretical foundation, recurring themes in the literature, and expert input. The scale utilized a five-point Likert-type scale, ranging from 5 (strongly agree) to 1 (strongly disagree).

#### Item revision through expert panel

There was a total of six subject matter experts that evaluated the 163-item pool for the ACN: SDH (See Table 1). The expert panel members included a diverse group of participants that were recruited by the author based on their known expertise in health disparities/SDOH research or practice (i.e., three or more publications in the area), behavioral/medical health practice with populations at risk for increased SDOH (three or more years of clinical practice with population), and quantitative methodology expertise (self-identified). The panel members were provided the theoretical framework for the scale and instructions that requested their decisions be informed by their expertise, the theoretical framework, and completed independently.

**Table 1:** Expert panel members characteristics

Reviewer	Race	Sex	Years of Experience	Current Position	Rounds
R1	White	F	15	Associate MD Director	1, 2, & 3
R2	Latinx	M	7	Assistant Professor	1, 2, & 3
R3	AA	M	10	Assistant Professor	1
R4	AA	M	5	Research Associate	1, 2, & 3
R5	AA	F	8	Assistant Professor	1, 2, & 3
R6	AA	F	30 +	Tenured Professor	2

*AA* African American

The subject matter experts who agreed to participate received a secured web link to the scale that included the 163 items and open-ended questions for feedback on individual items. Each reviewer worked independently and provided feedback for each item on the scale, including a rating of 1 = Keep item, 2 = Modify item, or 3 = Delete item. The ratings were also an indicator of the quality of the item, with one being the highest quality in terms of clarity, readability, and relatedness to the overall scale. Raters used an excel document to rate each item and provide feedback, with an average review time of eight days. Three expert panel review rounds resulted in 45 items and 94% agreement on the retained items in the ACN: SDH scale.

### Measures

#### The Addressing Client Needs with SDH scale (ACN: SDH)

Best practice guidelines for scale development were followed [25]. The scale included items that covered SDOH self-awareness and attitudes, knowledge, skills, and behaviors. The items are scored on a 5-point Likert scale, with 5-strongly agree, 4-agree, 3-neither agree nor disagree, 2-disagree, and 1-strongly disagree. Lower scores indicate less SDOH competency. Study 1 included 45 items, those items were reduced, and Study 2 included 22 items. Example questions include, “I am knowledgeable about the relationship between social determinants of health and behavioral health” and “It is not my role to address SDOH in practice”. Access to the full scale is included in a Supplementary file connected to this manuscript.

#### Healthcare Workers Cultural Competency (EMCC) [26]

This scale is a 14-item instrument designed to measure healthcare workers’ cultural competence. The scale has three subscales: sensitivity to own prejudices, cultural knowledge, and skills to work in culturally diverse environments. Responses are collected using five response categories: 1-Totally disagree, 2-disagree, 3- neither agree nor disagree, 4-agree, and 5-totally agree. Example questions include, “I believe patients’ beliefs, values, and customs affect their health” and “I am able to set therapeutic goals and/or objectives considering the cultural context (beliefs and customs) of my patients and their needs”. This scale was used in Study 2 to test the convergent and discriminant validity, and it had a Cronbach alpha of 0.90.

### Participants

Study 1 was completed by 220 participants; full results are in Table 2. In summary, the largest responses were in the following categories: Females 78.6% (*n* = 173), White 66.4% (*n* = 146), residing in the Southeast region of the United States 29.1% (*n* = 64), and identifying as mental/behavioral health service professionals 63.2% (*n* = 139). Study 2 was completed by 303 participants; full results are in Table 1. In summary, the largest responses were in the following categories: Females 80.5% (*n* = 244), White 52.8% (*n* = 160), residing in the Southeast region of the United States 40.9% (*n* = 124), and primarily identifying as a mental/behavioral health provider 50.2% (*n* = 152).

**Table 2:** Demographics for study 1 and study 2

	**Study 1 (*N* = 220)**		**Study 2 (*N* = 303)**	
	N	%	N	%
**Gender**				
Female	173	79	244	81
Male	44	20	54	18
Non-Binary	3	1	5	2
**Racial-Ethnic Identity**				
Asian	7	3	8	3
African American/Black	32	15	85	28
Hispanic/Latinx	30	14	35	12
White	246	66	160	53
Multiracial	5	2	12	4
Native Hawaiian/Pacific Islander/American Indian	-	-	3	1
**Region of the United States**				
Northeast	49	22	61	20
Southwest	42	19	47	16
West	30	14	31	10
Southeast	64	29	124	41
Midwest	30	14	30	10
Unknown	-	-	10	3
**Interprofessional Group of Health Care Providers**				
Mental/Behavioral Health Providers	139	63	152	50
Medical Health Providers	23	11	93	31
Healthcare Educators	48	21	39	13
Other Primary Work Industry	10	5	18	6

### Data analysis

The 45 items of the ACN: SDH scale were subjected to principal components analysis (PCA), and variable reduction technique, using SPSS version 18. Prior to performing the PCA, the suitability of the data for factor analysis was assessed. Confirmatory factor analysis (CFA), a statistical procedure to test how well the measured variables represent the constructs, was conducted using maximum likelihood estimation as implemented in SPSS 28 AMOS. Maximum likelihood with robust standard errors (MLR) was used to estimate model parameters and the goodness-of-fit of all the CFA models was examined with: The root-mean-square-error of approximation (RMSEA) ≤ 0.06 (90% CI ≤ 0.06), standardized root mean square (SRMR) ≤ 0.08, comparative fit index (CFI) ≥ 0.90, and tucker-lewis index (TLI) ≥ 0.90 [27, 28]. Additionally, the chi-square/df ratio ≤ 3 rule was also used [29].

## Results

### Study 1. Exploratory factor analysis and initial reliability

The suitability of the data was assessed through the following: Inspection of the correlation matrix revealed the presence of many coefficients of 0.30 and above. The Kaiser–Meyer–Olkin value was 0.92, exceeding the recommended value of 0.60 [30] and Bartlett’s Test of Sphericity [31] reached statistical significance, supporting the factorability of the correlation matrix (t (χ2 (990) = 6960.55, *p* < 0.001). The data was suitable for factor analysis.

Principal components analysis (PCA), initially revealed the presence of 9 components with eigenvalues exceeding 1, explaining a total of 69% of the variance. Inspection of the scree plot revealed the presence of six components; however, a model with four and five components was examined using varimax and oblimin rotations of the factor loading matrix. The model with six components explained a total of 61% of the variance, and it was preferred because of: (a) theoretical relevance and (b) eigenvalues on the scree plot leveled off after six. The varimax solution was used in subsequent analyses because the component transformation matrix had small values (0.22) indicating that components are not highly correlated which is the underlying hypothesis of the varimax solution. Lastly, a total of 20 items were eliminated because they either did not contribute to a simple factor structure and failed to meet a minimum criterion of having primary factor loadings of 0.4 or above, along with no cross-loadings of 0.3 or above.

Finally, a PCA, using varimax rotation was used on the 25-item ACN: SDH scale constrained, and a 6-factor solution was revealed, explaining 61% of the variance. Factor 1 contained six items that represented *Action Toward Addressing SDOH* and accounted for 33.96% of the variance. Factor 2 contained six items that represented *SDOH Knowledge* and accounted for 8.01% of the variance. Factor 3 contained three items that represented *Negative Attitudes toward Addressing SDOH* and accounted for 5.86% of the variance. Factor 4 contained three items that represented *Systemic Accountability* and accounted for 5.35% of the variance. Factor 5 contained four items that represented the *Perception of the cause of SDOH* and accounted for 4.04% of the variance. Factor 6 contained three items that represented *School Preparation* and accounted for 4.19% of the variance.

### Reliability analysis

The Cronbach alpha reliability estimate for the 25-item total scale was 0.809. Item total correlations ranged from *r* = -0.28 to *r* = 0.88 with a mean item-total correlation of *r* = 0.25. The six subscales demonstrated the following satisfactory internal consistency estimates: Factor 1, (*a* = 0.84); Factor 2, *(a* = *0.92);* Factor 3, (*a* = 0.74); Factor 4, (*a* = 0.79); Factor 5, (*a* = 0.87); and Factor 6, (*a* = 0.37). The study 1 results showed that the 25-item SDH: ACN has six dimensions which are concordant with the theoretical model.

### Study 2. Confirmatory Factor Analysis (CFA)

The purpose of Study 2 was to confirm the dimensionality and evaluate the nomological network of the ACN: SDH. Table 3 shows the CFA model comparisons; in the first model, all 25 items were allowed to load on a single factor (SDH provider competence). The second model is a first order orthogonal model where all items are loaded on the defined dimensions from the EFA (i.e., the six factors) and these dimensions were not correlated. The third model is the same as the second model except the six factors were allowed to correlate (i.e., oblique). The fourth model is a second order model in which all items were allowed to load on their defined dimensions and all dimensions loaded on a second order factor of ‘SDH provider competence’. All 4 models showed poor model fit; three indicators were identified as having a modification index larger than 20 causing the poor model fit. The items deleted included AP3, AP4, and ATT9. The fifth model is a first order oblique model where all items are loaded on the defined dimensions (i.e., six factors); this model showed acceptable model fit. The fit indices for each model are presented in Table 4.

**Table 3:** Exploratory and confirmatory factor analysis matrix for the ACN: SDH provider competency scale

	**Component**						
	**1**	**2**	**3**	**4**	**5**	**6**	**CFA**
**Factor 1: Action Toward Addressing SDOH (AC 1–6)**							
**b4 I have reviewed policy to address SDOH**	**.752**	.112	.128	.102	.168	-.044	.819^*^
**a6 I am aware of national grants to support SDOH R, IN, P**	**.709**	.190	-.045	-.018	-.057	-.037	.594^*^
**a7 I am aware of a questionnaire I can use to screen a client for SDOH needs**	**.683**	.027	.087	.152	.138	-.011	.643^*^
**b5 I have built relationships with community partners to address SDOH**	**.659**	.114	.230	.012	.136	.087	.675^*^
**s4 I am competent in tracking a clients health outcomes that are influenced by SDOH**	**.647**	.161	.165	.089	.258	.118	.775^*^
**b3 I have used the SDOH framework to address SDOH with clients**	**.624**	.071	.252	.173	.213	.044	**.**661
**Factor 2: SDOH Knowledge (KN 7–12)**							
**k4 I am knowledgeable about the relationship b/w SDOH & Behav. Health**	.105	**.791**	.290	.132	.154	.082	.839^*^
**k2 I am knowledgeable about the relationship b/w SDOH & health**	.182	**.782**	.166	.134	.115	.010	.865^*^
**k3 I am knowledgeable about the relationship b/w SDOH & chronic diseases**	.264	**.779**	.106	.180	.043	-.003	.840^*^
**k6 I am knowledgeable about the relationship b/w SDOH & health promotion**	.257	**.775**	.133	.208	.060	.025	.903^*^
**k1 I am knowledgeable about the drivers of health and mental health**	.068	**.774**	.155	.165	.167	.017	.880^*^
**k7 I am knowledgeable about the relationship between SDOH and resiliency**	.314	**.719**	.168	.084	.104	-.006	.841
**Factor 3: Negative Attitude toward Addressing SDOH (NA 13–15)**							
**att2r It is not my role to address SDOH in practice**^a^	.091	.059	**.726**	.083	-.095	-.152	.921^*^
**att7r Unmet SDOH needs does not impact the work I do with clients**^a^	-.098	.115	**.639**	.115	-.182	-.146	.859^*^
**att1r It is not practical for me to address SDOH in practice**^a^	.208	.022	**.612**	.010	-.045	-.131	.515
**Factor 4: Systemic Accountability (SA 16–18)**							
**att9 I think SDOH should be addressed at the societal level (i.e. policy)**	.064	.350	.116	**.738**	.054	-.015	––
**ap1 The shortage of government assistance is a cause of adverse SDOH**	.209	-.012	-.032	**.735**	.068	-.038	.779^*^
**ap2 Unequal educational opportunities is a cause of adverse SDOH**	.103	.073	.129	**.766**	-.015	-.054	.884
**Factor 5: Perception of the cause of SDOH (PC 19–22)**							
**ap3r Irresponsible behavior on the part of individual people is a cause of adverse SDOH**^a^	.098	-.055	.178	.355	**-.707**	-.162	––
**ap4r Poor decision making on the part of individual people is a cause of adverse SDOH**^a^	.080	-.018	.205	.343	**-.738**	-.169	**––**
**ap5 Mental illness and or substance abuse is a cause of adverse SDOH**	.212	.000	.090	.187	**.776**	-.212	.950^*^
**ap6 Physical illness and disability is a cause of adverse SDOH**	.226	.009	.129	.316	**.728**	-.172	.932
**Factor 6: Preparation (WK 23–25)**							
**p2 Practicum and internship prepared me to address SDOH in practice**	.198	.152	.053	.061	-.056	**.826**	.825^*^
**p1 The coursework in my program prepared me to address SDOH in practice**	.233	.191	-.022	.003	-.029	**.791**	.844^*^
**p3 Interprofessional collaboration training during my degree program**	.303	.074	-.006	.105	-.002	**.743**	.803

Bold values indicate the highest factor loading for each item; CFA factor loadings significant at the *p* <.01 = ^*^

^a^Reverse-scored item = r

**Table 4:** Fit indices for confirmatory factor analysis of the ACN: SDH

Model	X^2^	*df*	CFI	RMSEA (90%CI)	SRMR
Model 1: One factor model	2947.2	275	.454	.17 [.161, .178]	.146
Model 2: First-order orthogonal six factor model	1071.9	260	.834	.10 [.095, .108]	.086
Model 3: First order oblique six factor model	1101.8	269	.830	.10 [.095, .108]	.092
Model 4: Second order factor model	1223.8	274	.806	.10 [.101, .113]	.163
Model 5: First-order oblique six factor model (22 items)	460.3	194	.937	.06 [.059, .075]	.048

*ACN:SDH* Social Determinants of Health Provider Competency Scale, *CFI* Comparative fit index, *RMSEA* Root mean square error of approximation, *90% CI* 90% Confidence Interval for RMSEA (lower limit, upper limit), *SRMR* Standardized root mean square residual

Model 5 first order oblique 6-factor model showed a better fit than the other models. Fitting the oblique model indicates that specific domains of provider SDOH competency are not interrelated. The chi square, CFI, RMSEA, and SRMR values indicate good to reasonable fit of model 5 (χ2 = 460.281, df = 194, CFI = 0.937, RMSEA = 0.067, SRMR = 0.048). The factor loadings for the preferred first order model (i.e., model 5) are presented in Table 4.

### Reliability analysis

Internal consistency estimates of the ACN: SDH total score and subscale scores for Study 2 were high. The Cronbach alpha reliability estimate for the 22-item total score was 0.867. Item total correlations ranged from *r* = -0.27 to *r* = 0.89 with a mean item-total correlation of *r* = 0.23. The six subscales demonstrated satisfactory internal consistency estimates, listed in Table 4: Factor 1, (*a* = 0.85); Factor 2, *(a* = 0.94*);* Factor 3, (*a* = 0.79); Factor 4, (*a* = 0.81); Factor 5, (*a* = 0.86); and Factor 6, (*a* = 0.94). The total 22 item scale demonstrated satisfactory internal consistency. Study 2 results indicate that the 22-item ACN: SDH has six dimensions which is concordant with the theoretical model.

#### Scoring

The ACN: SDH items are scored on a scale from 1 to 5, with items with an ‘r’ being reversed-coded. The mean scores and standard deviations for the total 22 item ACN: SDH scale and subscale scores are presented in Table 5. Lower scores indicate less competency.

**Table 5:** Descriptive statistics and alpha for 22 item ACN: SDH (Study 2)

	Mean	Min	Max	SD	Cronbach alpha	EMCC Correlation
Measure						
ACN:SDH total scale	77.73	42	102	11.46	.87	.47^a^
AC	18.89	6	30	4.99	.85	.29^a^
KN	25.05	6	30	4.74	.94	.41^a^
NA	12.05	3	15	2.58	.79	.39^a^
SA	7.85	2	10	1.78	.81	.44^a^
PC	4.24	2	10	1.81	.94	-.26^a^
WK	9.63	3	15	2.98	.86	.24^a^

^a^correlation is significant at the 0.01 level (2-tailed); Six subscales = *AC* Action toward addressing SDOH, *KN* Sdoh knowledge, *NA* Negative attitude toward addressing sdoh, *SA* Systemic accountability, *PC* Perception of the cause of sdoh, & *WK* School preparation

### Convergent and discriminant validity

Pearson’s correlations between the ACN: SDH and the EMCC scales are summarized in Table 5. The ACN: SDH total and six subscale scores showed a low to moderate positive correlation to all subscales that are conceptually similar in content, which shows convergent validity. Discriminant validity is indicated by the inverse relationship between the EMCC and the ACN: SDH Perception subscale’ which is hypothesized to be conceptually different.

### Factor Invariance Analysis (FIA)

Confirmatory factor analysis was used to test whether the ACN: SDH questionnaire is equivalent among the healthcare provider groups (i.e., Behavioral/Mental health & Medical health); 4-factor models were estimated (i.e., configural, metric, scalar, and strict) and The Chi-square difference test was conducted to determine metric invariance. In terms of configural invariance, the ACN:SDH scale maintained the same number of dimensions in each group. The factor loadings and the item means were similar between the groups, supporting both metric and scalar invariance. Lastly, a chi-square difference test was conducted, which tests whether the model represents a significantly worse fit to the data than the previous model (assuming configural invariance) and the *p*-value is 0.206, which is not significant, interpreted to mean that the metric invariance model holds.

### Subgroup analysis

Subgroup analysis was conducted using ANOVA. A one-way between group analysis of variance was conducted to explore ‘healthcare provider industry’ on levels of SDH competency, as measured by the ACN: SDH. Participants were divided into four groups according to their healthcare provider industry (Group 1: Behavioral/Mental health, *n* = 152; Group 2: Medical health services, *n* = 93; Group 3: Educators, *n* = 39; Group 4: Other, *n* = 18). There was a statistically significant difference at the *p* < 0.01 level in ACN: SDH scores for the four groups: F (3, 301) = 6.499, *p* = 0.001. The effect size calculated using eta squared, was 0.06, which is a medium effect size [32]. Post-hoc comparisons using Tukey HSD test indicated that the mean score for the Behavioral/Mental health group (*M* = 80.03, *SD* = 10.80) was statistically different from that of the Medical Health Services (*M* = 76.09, *SD* = 11.49) and Other group (*M* = 69.11, *SD* = 14.04). The Education group (*M* = 76.67, *SD* = 10.40) did not differ significantly from either group.

## Discussion

The EFA (study 1) and the CFA (study 2) results supported a six-dimension SDOH provider competency scale with a total of 22 items. In addition, the total scale and the subscales showed good reliability estimates all between 0.79 and 0.94, as seen in Table 5. The scale showed good convergent and discriminant validity, interpreted to mean that the scale is measuring what it is intended to measure (i.e., SDOH competency). In addition, measurement invariance was conducted (i.e., FIA) and results suggested that the scale can be used with different healthcare provider groups, making this a highly usable and flexible measure of health service professionals’ competency. It was hypothesized that the scale would measure similar constructs across health service professionals’ because the underlying values of health service professionals are similar, such as patient empowerment and patient-centered care [33].

The subgroup analysis, using ANOVA, was used to determine health service professionals’ competency with SDOH. SDOH competence includes awareness, knowledge, and having the skills to address the needs in practice or at your level of influence (i.e., civic engagement, policy, advocacy) [6, 8]. Prior research shows that without provider competence SDOH needs fail to be addressed in practice [6, 8]. The participants included in the study all showed acceptable SDOH competence, scoring above or at the mean.

In terms of theoretical alignment, the six dimensions of the scale are aligned with the theoretical foundation of the multicultural counseling competencies [24] and the Institute of Medicine’s framework for understanding collaborative practice and patient outcomes [34]. The theoretical foundation would indicate that competency should be examined from multiple dimensions and the ACN: SDH includes six factors representing competency through, action (factor 1), knowledge (factor 2), attitudes (factor 3), accountability (factor 4), school preparation (factor 5), and perceptions (factor 6). These findings collectively support the validity of interpretations and inferences that can be made from the scores on the ACN: SDH scale.

In addition to the results, there are a few limitations. The scale is validated in a subpopulation of healthcare workers in the United States, which may limit the generalizability of the scale to those outside of the United States and among other subpopulations of healthcare workers; researchers should take care of this limitation when utilizing the scale and confirm the reliability of the scale with their unique population. There is also the potential for selection bias. It is assumed that health service professionals who completed the study voluntarily have some level of care or interest in addressing SDOH challenges. This bias toward addressing the issue could potentially lead to social desirability responses. Future research studies are needed to contribute more evidence of construct validity and to establish test–retest reliability. Additionally, research can seek to understand if some SDOH competence domains have different levels of influence as it relates to competence and outcomes (i.e., weighting the SDOH dimensions). Lastly, research can also focus on continued tests of measurement invariance for different healthcare provider groups or by other variables that can impact provider SDOH competency.

### Implications

#### Health service professionals

The ACN:SDH scale, is an SDOH competency scale for health service professionals. Professionals on the frontline of healthcare service delivery, are encouraged to be competent in addressing SDOH challenges systemically and at the individual level. The ACN: SDH scale is an opportunity to self-assess competence for addressing SDOH systemically and with individual patients. Completing a competence self-assessment creates space for self-reflection and developing a plan for growth. The hope is that health service professionals will take the ACN: SDH, explore their areas of growth and then develop an action plan to gain more competence through professional development and training. For example, after a health service professional completes the ACN: SDH, they can consult with colleagues and form a community of practice that supports each other in developing SDOH competence, through shared learning, expertise, and resources, as well as engaging in professional development activities. The community of practice might also consider taking the ACN: SDH at two-time points to track their growth. There are many opportunities and ways to use the ACN: SDH tool and for the healthcare field, it provides a standardized measure to address SDOH competence in its totality.

#### Healthcare system

As a system, it is important to support the development of a workforce that is knowledgeable and capable of addressing SDOH systemically and at the individual patient levels [35]. A healthcare system could deploy the ACN: SDH tool to all health service professionals in their system to understand the gaps in provider competence. The results from the ACN: SDH can be utilized to create a strategic workforce development plan that includes benchmarks for growth related to the major gaps of competence highlighted. In addition, the system can develop a series of professional development and continuing education initiatives that seek to improve SDOH competence. Along with the direct consumer-level benefits the scale can also be used in research studies that seek to understand the relationships between SDOH competency and care outcomes, which are especially important right now amid a Covid-19 recovery effort. Society and its members are increasingly faced with more economic and social health challenges than before, and it is imperative that the workforce is prepared to manage and address these issues with care and competence.

## Conclusion

This study reports on the development and validation of an instrument (i.e., ACN: SDH) to measure health service professionals’ competence in addressing SDOH challenges in practice. The results support the validity, reliability, and high comparability between health service professionals’ groups. The current study contributes to the literature on health service professionals’ competence in addressing SDOH challenges in practice and provides healthcare service professionals a tool for self-assessment of SDOH competence; as well as providing healthcare systems a flexible and valid tool for assessing their workforce and addressing continuing education needs.

## Supplementary Information

**Additional file 1.**

## Data Availability

The datasets analyzed during the current study are available from the corresponding author on reasonable request.

## Abbreviations

SDOH: Social determinants of health
EFA: Exploratory factor analysis
CFA: Confirmatory factor analysis
ACN: SDH: The addressing client needs with social determinants of health scale
EMCC: Healthcare workers cultural competency
