# Supplementary material for: Development and initial validation of the addressing client needs with social determinants of health scale (ACN: SDH)
Source: BMC Health Serv Res. 2023 Apr 19;23:374. doi: 10.1186/s12913-023-09292-z (PMC10113720; doi:10.1186/s12913-023-09292-z)
Supplement: Supplementary file 1 — Additional file 1. [file 12913_2023_9292_MOESM1_ESM.docx]

**The Addressing Client Needs with SDH survey Items (ACN: SDH)**

**Instructions:** The scale seeks to understand areas of growth related to social determinant of health (SDOH) competence and has no right or wrong answers. The scale is 22 brief statements that prompt you to respond using a Likert type scale from 1-strongly disagree to 5-strongly agree.

**Action Toward Addressing SDOH**

1. **b4 I have reviewed policy to address SDOH**
2. **a6 I am aware of national grants to support SDOH research, interventions, or practice**
3. **a7 I am aware of a questionnaire I can use to screen a client for SDOH needs**
4. **b5 I have built relationships with community partners to address SDOH**
5. **s4 I am competent in tracking a clients health outcomes that are influenced by SDOH**
6. **b3 I have used the SDOH framework to address SDOH with clients**

**SDOH Knowledge**

1. **k4 I am knowledgeable about the relationship b/w SDOH and behavioral health**
2. **k2 I am knowledgeable about the relationship b/w SDOH and health**
3. **k3 I am knowledgeable about the relationship b/w SDOH and chronic diseases**
4. **k6 I am knowledgeable about the relationship b/w SDOH and health promotion**
5. **k1 I am knowledgeable about the drivers of health and mental health**
6. **k7 I am knowledgeable about the relationship between SDOH and resiliency**

**Attitude toward Addressing SDOH**

1. **att2r It is not my role to address SDOH in practice**
2. **att7r Unmet SDOH needs does not impact the work I do with clients**
3. **att1r It is not practical for me to address SDOH in practice**

**Systemic Accountability**

1. **ap1 The shortage of government assistance is a cause of adverse SDOH**
2. **ap2 Unequal educational opportunities is a cause of adverse SDOH**

**Perception of the cause of SDOH**

1. **ap5 Mental illness and or substance abuse is a cause of adverse SDOH**
2. **ap6 Physical illness and disability is a cause of adverse SDOH**

**Preparation**

1. **p2 Practicum and internship prepared me to address SDOH in practice**
2. **p1 The coursework in my program prepared me to address SDOH in practice**
3. **p3 Interprofessional collaboration training during my degree program**
